# Optimal design of agro-residue filled poly(lactic acid) biocomposites using an integrated CRITIC-CoCoSo multi-criteria decision-making approach

**DOI:** 10.1038/s41598-025-92724-z

**Published:** 2025-04-04

**Authors:** László Lendvai, Sándor Kálmán Jakab, Tej Singh

**Affiliations:** 1 Department of Materials Science and Engineering, Széchenyi István University, Gyor, 9026 Hungary; 2 Faculty of Informatics, Savaria Institute of Technology, Eötvös Loránd University, Budapest, 1117 Hungary

**Keywords:** Poly(lactic acid), Biocomposites, Circular economy, Agricultural waste, Decision making, CRITIC-CoCoSo, Engineering, Materials science, Mathematics and computing

## Abstract

In recent years, there has been a rise in environmental awareness, leading to increased efforts to develop eco-friendly materials as alternatives to petroleum-based polymers. This study examined the performance optimization of poly(lactic acid) (PLA) biocomposites filled with agricultural byproducts at concentrations ranging from 0 to 20% by weight, highlighting their potential as substitutes for commodity plastics. The agro-residues used as fillers were flax seed meal and rapeseed straw. A hybrid decision-making algorithm was proposed, utilizing the “criteria importance through inter-criteria correlation” (CRITIC) alongside the “combined compromise solution” (CoCoSo), aimed at identifying the optimal alternative among the evaluated samples. The algorithm considered several attributes, including mechanical traits evaluated via tensile, flexural, and impact tests, hardness, water absorption, biodegradation, and production cost. The findings revealed that the strength properties, including tensile, flexural, impact, and water absorption, were most advantageous for neat PLA. In contrast, the highest modulus values were recorded for the biocomposite filled with 20 wt% rapeseed straw. The biocomposites exhibit increased hardness as agro-waste content rose, with the highest hardness observed in the biocomposite filled with 20 wt% flax seed meal. The study on biodegradation indicates that a higher content of agro-waste promotes disintegration, with flax seed meal emerging as the most effective additive in this context. The findings show that adding various agricultural byproducts in varying amounts affects the evaluated properties differently. Hence, the hybrid CRITIC-CoCoSo optimization approach is utilized to choose the optimal biocomposite. The findings show that the biocomposite with 20 wt% rapeseed straw demonstrated optimal physico-mechanical and biodegradation properties, making it a promising eco-friendly alternative for future applications.

## Introduction

The past decades have seen an enhanced environmental awareness, primarily due to the massive increase in urbanization, which inherently came with an immense amount of waste production in line with the services provided to the public. In this respect, one of the most problematic topics is the issue of single-use plastic products that are fabricated by employing petroleum-derived polymeric materials with low degradation rates. In order to mitigate the problems brought by the accumulation of waste, the current literature is intensely focused on two significant topics: (i) large-scale recycling feasibilities of plastic products after the end of their useful life^1–3^ and (ii) substituting them with biodegradable alternatives that are preferentially derived from renewable (green) resources^4–7^. Both routes support the principles of circular economy. However, there are several challenges to overcome, which is a major driving force for research and development in these fields^8^.

One of the major obstacles for the latter route is the relatively low number of available bioplastics, and even those come at a cost greatly exceeding that of conventional polymeric materials^9^. A potential way to expand the possible fields of application for such biopolymers is to alter their properties. In the plastic industry, the most common approach to achieve property-tailored materials is to pair a polymer matrix with other polymers or various additives, thereby creating blends or composites. Preserving the green nature of the resulting binary or ternary polymeric materials dramatically limits the range of potential secondary and tertiary components. In this respect, employing natural fibers as reinforcement in polymer composites seems an optimal choice^10–12^. The primary benefit of natural fiber-reinforced polymer composites (NFRPCs) based on a biopolymer matrix is that they are entirely derived from renewable sources while also being biodegradable^13,14^. Natural fibers have numerous other advantages compared to their synthetic counterparts, including low density and cost, non-abrasive characteristics, high stiffness, and simpler processing^15^. A specific class of natural fibers is the agro-residues, which is a group of lignocellulosic particles that are generated as by-products of agricultural activities and which have been widely tested for filler purposes in biocomposites recently^16–19^. Such natural fillers/fibers include straws, stems, shells, and fibers of various plants. The applications of these fibers and fillers are shifting away from reliance on forest wood and synthetic fibers towards more sustainable, ecological alternatives. These natural fillers/fibers offer a low carbon footprint and are abundant, biodegradable, recyclable, and non-toxic^20,21^. Biocomposites made from agro-residues have contributed significantly to the rise of environmental consciousness and the establishment of a sustainable equilibrium between agricultural practices and industrial activities^21,22^. The availability of natural particles is plenty and considering their different properties, they offer a wide range of options for material designers to choose from.

Among bioplastics, poly(lactic acid) (PLA) is receiving significant attention. PLA is an aliphatic polyester that demonstrates favorable mechanical properties, optical transparency, and effective processability, positioning it as a viable alternative to various synthetic polymers such as polypropylene, polystyrene, and poly(ethylene terephthalate)^23^. Conversely, its intrinsic brittleness, inadequate gas barrier properties, and elevated production costs impede its widespread adoption^9^. Cost-related issues can be effectively addressed by integrating low-cost secondary components. This is the point, where lignocellulosic agricultural and forestry by-products have their *raison d’être*.

Liao et al.^24^ investigated the effect of rice straw fibers on PLA-based biocomposites. Based on their findings, incorporating rice straw fibers into the PLA matrix does not result in any enhancement of mechanical properties, albeit, acting as a nucleating agent, it effectively increased the crystallinity of the polymer. Yussuf et al.^25^ fabricated PLA/rice husk and PLA/kenaf biocomposites with 20 wt% filler content. Based on their findings, the rice husk and kenaf improved the flexural modulus of PLA (3.4 GPa) to 4.0 GPa and 4.5 GPa, respectively. Meanwhile, the polymer’s flexural strength and impact strength decreased slightly. Kenaf outperformed rice husk as filler for all examined mechanical parameters. The authors also found that adding natural fibers slightly improves the biodegradability of PLA, with kenaf having a more significant effect on the biodegradation rate. Finkenstadt and colleagues^26^ prepared PLA-based biocomposites using various oilseed co-products, such as Cuphea, Lesquerella, and milkweed. The amount of fillers incorporated into the PLA was in the range of 0–45 wt%. The authors revealed that with growing co-product content, the tensile strength of PLA decreased consistently with the Nicolais-Narkis model. Cuphea was shown to improve the stiffness of the polymer, unlike Lesquerella and milkweed, which both decreased it slightly. Unexpectedly, the milkweed-filled samples showed extensive stress-cracking under tensile load and exhibited a 50–200% elongation greater than unfilled PLA.

The impact of wood particles on the mechanical characteristics of PLA composites fabricated by 3D printing was studied by Kariz et al.^27^ from 0 to 50% wt%. It was observed that the density of the composites reduced little as the wood component increased. After adding 10 wt% wood particles, the tensile strength of the composites rose from 55 MPa to 57 MPa and then dropped with further addition of wood particles. A relative increase of around 20% in tensile modulus was found for composites loaded with 20 wt% wood particles, which after that dropped. The effects of adding marine and agricultural waste in 3D-printed PLA biocomposites were studied by Scaffaro et al.^28^. Experiments were carried out to investigate the impact of varying waste concentrations (10 and 20 by weight) on the mechanical characteristics of PLA biocomposites. The findings demonstrated that the assessed mechanical properties noted a decline with increasing waste content. In contrast, biocomposites containing 10 wt% marine and agricultural waste particles were shown to have a relative improvement in impact strength of 14% and 20% when compared to unfilled PLA.

Scaffaro et al.^29^ also examined how variations in particle size (< 150 μm and 150–300 μm) and the quantity of lignocellulosic flour from *Opuntia ficus indica* (10 and 20 wt%) affected the mechanical performance of PLA-based composites. According to the authors, the tensile strength and elongation at break of PLA decreased as the lignocellulosic flour content rose. The tensile modulus, in contrast, rose by 36% and 25% compared to unfilled PLA for lignocellulosic flour-filled composites ranging in size from 150 to 300 μm, respectively. The mechanical characteristics of wood flour/natural cork hybrid filler (ranging from 0 to 30% by weight) filled PLA-based composites was thoroughly examined by Andrzejewski et al.^30^. The authors found that adding fillers to the composites reduced their strength performance. However, they found that the composites’ tensile modulus significantly improved, particularly with PLA composites that included 30 wt% wood flour. A recent work conducted by Huang et al.^31^ sought to investigate how biochar derived from grapevine crop residue affected the mechanical characteristics of PLA composites when added at concentrations of 1 wt% and 10 wt% with particle sizes of 100 and 200 mesh, respectively. The analysis revealed that the impact and tensile strengths of PLA composites increased at 1 wt% filler particles for both particle sizes prior to failure, while the Young’s modulus exhibited a continuous rise with higher filler concentrations.

According to the literature, lignocellulosic filler may improve mechanical performance, decrease weight, and boost biodegradability. For composites to be designed successfully, several interrelated and, at times, competing performance criteria are required. Furthermore, differently produced composites exhibited varying performance in the investigated attributes, necessitating a judgment on the optimal composite that best fulfills all performance parameters. One way to do this is using multi-criteria decision-making (MCDM) analysis. Therefore, the composite designers have proposed several methodologies to determine the best composite design, including AHP, CRITIC, PROMETHEE, TOPSIS, MOORA, EDAS, TODIM, VIKOR and many more^32–35^. However, several of these approaches need additional parameters for computations, and some of them are time-consuming due to their large computing requirements^36,37^. Thus, a straightforward technique is still required to handle formulation optimization difficulties. In contrast, Yazdani et al.^38^ introduced a combined compromise solution (CoCoSo) technique, which is straightforward and does not need complex calculations. The CoCoSo methodology was successfully applied in the selection of engineering sustainability components by Dwivedi and Sharma^39^, in assessing the accessibility of urban green spaces and parks by Ghasemiet al.^40^, in supply chain management by Jafari and Khanachah^41^, and in evaluating anti-tank guided missiles by Erdal et al.^42^. In addition, Peng et al.^43^ used the combined CRITIC-CoCoSo approach to assess the 5G sector, Negara et al.^44^ used it in named data networking, Gou^45^ utilized it for quality assessment of entrepreneurship education and innovation in vocational institutions, and Nguyen and Chaysiri^46^ implemented it to determine the optimal location for solar-wind energy plants.

As highlighted above, the different MCDM techniques have been widely used in various fields over the last several years^47–49^. However, there is a lack of literature on applying these methods in biopolymer-based natural fiber composites. Existing research mainly focuses on investigating these novel materials’ mechanical, physical, thermal, and other properties without examining their overall potential through complex systematic decision-making frameworks. This gap highlights the necessity for scientific works to explore the possibility of such techniques in the field of natural fiber based composites. Therefore, in this work, agricultural by-product-filled PLA biocomposites, previously developed and assessed for thermal, physical, and mechanical performance in a prior study, are utilized^50^. Given the challenge of selecting the most suitable biocomposite from the array of manufactured and mechanically evaluated options, a hybrid CRITIC-CoCoSo-based MCDM approach is proposed. This method determines the optimal candidate that best balances the conflicting properties evaluated within the composites. The main contribution of this work is developing a hybrid CRITIC-CoCoSo-based MCDM model for selecting the best PLA biocomposite material filled with agricultural byproducts among several competing criteria. Sensitivity analysis and a comparison with other MCDM models were used to further investigate the robustness and consistency of the proposed hybrid CRITIC-CoCoSo approach.

The study is structured as follows: Section “Optimization methodology” discusses the used optimization methodology. Section “Alternatives and criteria selection” discusses the selection of biocomposite alternatives and the assessment criteria. Section “Results and discussion” presents the ranking results and sensitivity analysis. Conclusions are presented in Section “Conclusions”.

## Optimization methodology

**Fig. 1:**
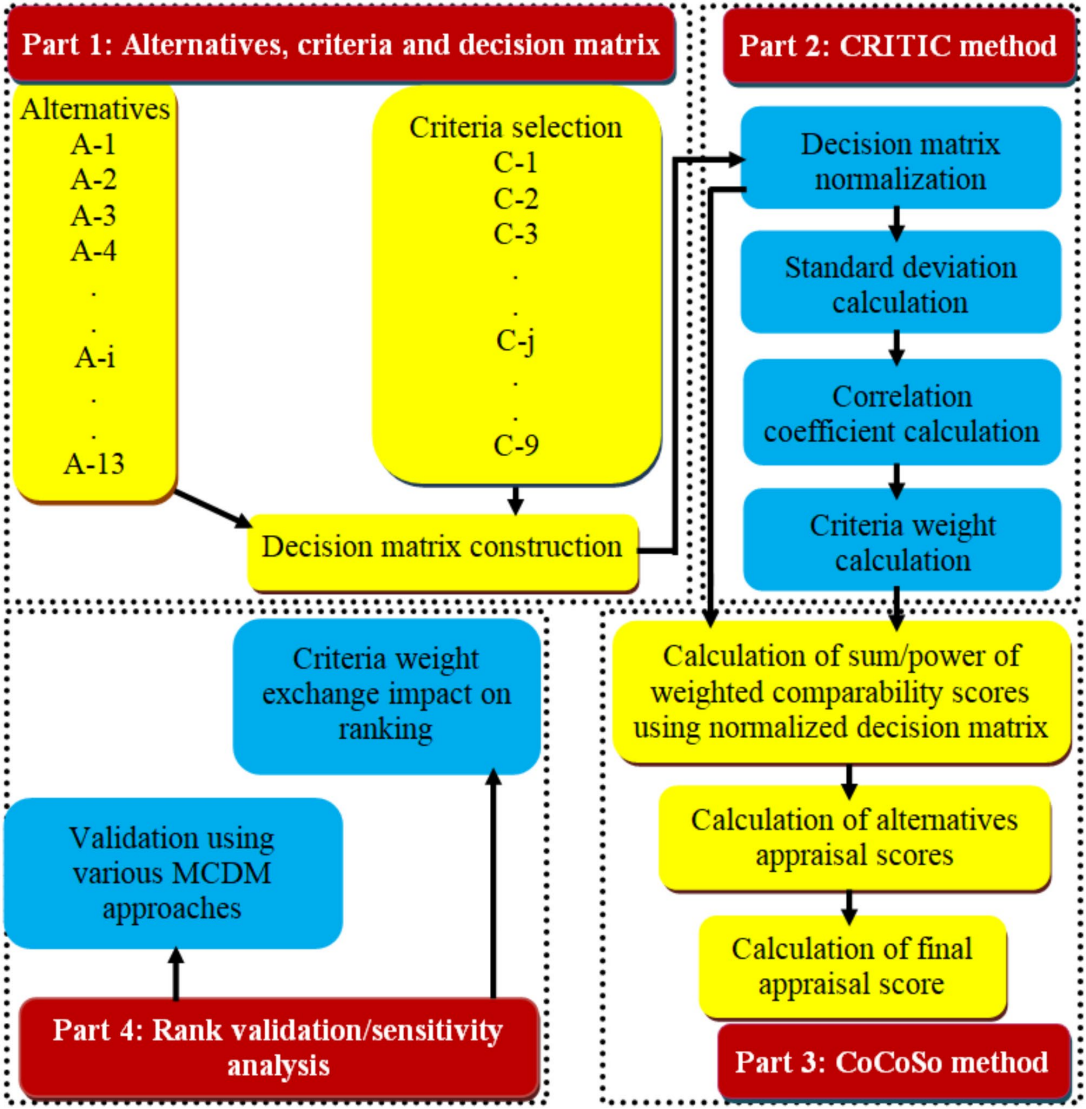
The architecture of the proposed optimization methodology.

This study aims to evaluate and rank biocomposite alternatives based on assessment criteria related to their properties, utilizing a hybrid MCDM approach that combines CRITIC and CoCoSo methods. The CoCoSo method ranks the biocomposites alternatives, whereas the CRITIC method measures the criteria weights. The procedure for CRITIC-CoCoSo analysis has been described in three parts and it was followed by sensitivity analysis and validation as presented in Fig. 1.

*Part 1*: Alternatives, criteria, and decision matrix.

In the first step, the alternatives and criteria for a specific MCDM problem are defined. The stated alternatives and criteria are then described as a decision matrix (DM). In general, Eq. 1 shows how to create a DM for any MCDM problem with m alternatives $\left( {{A_i},i=1,2, \ldots ,m} \right)$ and n criteria $\left( {{C_j},j=1,2, \ldots ,n} \right)$.

1 $$DM={\left[ {{d_{ij}}} \right]_{m \times n}}=\begin{array}{*{20}{c}} \begin{gathered} {A_1} \hfill \\ {A_2} \hfill \\ \vdots \hfill \\ \end{gathered} \\ {{A_i}} \\ \vdots \\ {{A_m}} \end{array}\mathop {\left[ {\begin{array}{*{20}{c}} {{d_{11}}}&{{d_{12}}}& \ldots &{{d_{1j}}}& \ldots &{{d_{1n}}} \\ {{d_{21}}}&{{d_{22}}}& \ldots &{{d_{2j}}}& \ldots &{{d_{2n}}} \\ \vdots & \vdots & \ldots & \vdots & \ldots & \vdots \\ {{d_{i1}}}&{{d_{i2}}}& \ldots &{{d_{ij}}}& \ldots &{{d_{in}}} \\ \vdots & \vdots & \ldots & \vdots & \ldots & \vdots \\ {{d_{m1}}}&{{d_{m2}}}& \ldots &{{d_{mj}}}& \ldots &{{d_{mn}}} \end{array}} \right]}\limits^{{\begin{array}{*{20}{c}} {{C_1}\;\;{\kern 1pt} \quad {C_2}\quad \cdots }&{{C_j}\;}& \cdots &{{C_n}} \end{array}}}$$

where an element ${d_{ij}}$of the decision matrix signifies the performance score of the $i{\text{th}}$alternative, ${A_i}$, with respect to the $j{\text{th}}$ criterion, ${C_j}$.

*Part 2*: CRITIC method.

The CRITIC method demonstrates extensive applicability across the fields of engineering and economics. This method is employed to determine the weights of criteria in the presence of experimental data. The procedure for performing calculations is outlined in the subsequent steps^43–46,51^.

*Step 1*: Decision matrix normalization (${\aleph _{ij}}$)

After the construction of decision matrix, the criteria values are normalized according to their implication. For higher-the-better (${n_h}$) and lower-the-better (${n_l}$) criteria the normalization is performed using Eqs. 2 and 3:

2 $${\aleph _{ij}}=\frac{{{d_{ij}} - d_{j}^{{\hbox{min} }}}}{{d_{j}^{{\hbox{max} }} - d_{j}^{{\hbox{min} }}}}\quad if\;j \in {n_h}$$

3 $${\aleph _{ij}}=\frac{{d_{j}^{{\hbox{max} }} - {d_{ij}}}}{{d_{j}^{{\hbox{max} }} - d_{j}^{{\hbox{min} }}}}\quad if\;j \in {n_l}$$

*Step 2*: Correlation coefficient calculation (${\chi _{j\lambda }}$)

After performing normalization, the correlation coefficient (${\chi _{j\lambda }}$) between the criteria is determined. For the *j*-th and *λ*-th criteria, the ${\chi _{j\lambda }}$calculated as described in Eq. 4.

4 $${\chi _{j\lambda }}=\frac{{\sum\limits_{{i=1}}^{m} {\left( {{\aleph _{ij}} - \overline {{{\aleph _j}}} } \right)\left( {{\aleph _{i\lambda }} - \overline {{{\chi _\lambda }}} } \right)} }}{{\sqrt {\sum\limits_{{i=1}}^{m} {{{\left( {{\aleph _{ij}} - \overline {{{\aleph _j}}} } \right)}^2}\sum\limits_{{i=1}}^{m} {{{\left( {{\aleph _{i\lambda }} - \overline {{{\aleph _\lambda }}} } \right)}^2}} } } }}$$

where the mean of the *j*-th and *λ*-th criteria are represented by $\overline {{{\aleph _j}}}$ and $\overline {{{\aleph _\lambda }}}$. Equation 5 is used for $\overline {{{\aleph _j}}}$ calculation.

5 $$\overline {{{\chi _j}}} =\frac{1}{n}\aleph \sum\limits_{{j=1}}^{n} {{\chi _{ij}};\quad i=1, \ldots ,m}$$

Similarly, by replacing *j* with *λ* in Eq. 4 was used to calculate$\overline {{{\chi _\lambda }}}$.

*Step 3*: Standard deviation calculation (${\rho _j}$)

In this step, the standard deviation (${\rho _j}$) for various criteria is determined using the formula in Eq. 6.

6 $${\rho _j}=\sqrt {\frac{1}{{n - 1}}\sum\limits_{{j=1}}^{n} {{{\left( {{\aleph _{ij}} - \overline {{{\aleph _j}}} } \right)}^2}} } ;\quad i=1, \ldots ,m$$

*Step 4*: Index value calculation (${\Delta _j}$)

For the selected criteria, the index value (${\Delta _j}$) computed using Eq. 7.

7 $${\Delta _j}={\rho _j}\sum\nolimits_{{j=1}}^{n} {\left( {1 - {\chi _{j\lambda }}} \right)}$$

*Step 5*: Criteria weights calculation (${\omega _j}$)

The weights of attributes are determined as described in Eq. 8.

8 $${\omega _j}=\frac{{{\Delta _j}}}{{\sum\nolimits_{{j=1}}^{n} {{\Delta _j}} }}$$

*Part 3*: CoCoSo method.

The CoCoSo method, first introduced by Yazdani et al.^38^, is an MCDM technique that has been successfully applied to solve various types of complex decision-making problems. Using the CoCoSo method the decision problem is solved in the following steps^38,43–46^.

*Step 1*: First of all, a DM showing the performance of different alternatives with respect to various criteria is constructed as presented in Eq. 1.

*Step 2*: The DM contains different entities and to make them comparable, a normalization procedure is implemented according to higher-is-better (${n_h}$) and lower-is-better (${n_l}$) criteria implication as presented in Eqs. 2 and 3.

*Step 3*: After normalization, the weighted sum comparability score (${\varpi _{ij}}$) and weighted power comparability score ($\varpi _{{ij}}^{ * }$) of alternatives are determined using Eqs. 9 and 10.

9 $${\varpi _{ij}}=\sum\nolimits_{{j=1}}^{n} {{\aleph _{ij}}{\omega _j}}$$

10 $$\varpi _{{ij}}^{ * }=\sum\nolimits_{{j=1}}^{n} {{{\left( {{\aleph _{ij}}} \right)}^{{\omega _j}}}}$$

*Step 4*: In this step, appraisal score strategies are applied to produce the relative weights of the alternatives using three relations. The first relation is the arithmetic mean of sums (${\Re _{ia}}$) of ${\varpi _{ij}}$ and $\varpi _{{ij}}^{ * }$expressed using Eq. 11, while the second relation is the sum of relative scores (${\Re _{ib}}$) of ${\varpi _{ij}}$ and $\varpi _{{ij}}^{ * }$ determined using Eq. 12. The third relation is the balanced compromised solution (${\Re _{ic}}$) between ${\varpi _{ij}}$ and $\varpi _{{ij}}^{ * }$ computed using Eq. 13.

11 $${\Re _{ia}}=\frac{{{\varpi _i}+\varpi _{i}^{ * }}}{{\sum\nolimits_{{i=1}}^{m} {\left( {{\varpi _i}+\varpi _{i}^{ * }} \right)} }}$$

12 $${\Re _{ib}}=\frac{{{\varpi _i}}}{{\mathop {\hbox{min} }\limits_{i} {\varpi _i}}}+\frac{{\varpi _{i}^{ * }}}{{\mathop {\hbox{min} }\limits_{i} \varpi _{i}^{ * }}}$$

13 $${\Re _{ic}}=\frac{{\chi ({\varpi _i})+(1 - \chi )\varpi _{i}^{ * }}}{{\chi \mathop {\hbox{max} }\limits_{i} {\varpi _i}+(1 - \chi )\mathop {\hbox{max} }\limits_{i} \varpi _{i}^{ * }}};\quad 0 \leqslant \chi \leqslant 1\quad$$

In Eq. 13, $\chi$ = 0.5, and its selection is based on the decision-makers.

*Step 5*: Finally, alternatives are ranked according to their performance factor (${\Re _i}$) determined using Eq. 14. The alternative with the highest ${\Re _i}$value is the most preferred one.

14 $${\Re _i}={({\Re _{ia}} \times {\Re _{ib}} \times {\Re _{ic}})^{\frac{1}{3}}}+\frac{1}{3}\left( {{\Re _{ia}}+{\Re _{ib}}+{\Re _{ic}}} \right)$$

*Part 4*: Rank validation and sensitivity analysis.

To validate the outcomes of the suggested approach, a comparison study was performed by comparing the rankings of alternatives obtained from many well-established MCDM approaches, such as MABAC^52^, MAUT^53^, ROV^54^, and MACBETH^55^. The criterion weights were switched throughout the sensitivity study, and for every alteration, a fresh ranking analysis was carried out using the CoCoSo technique.

## Alternatives and criteria selection

### Composite fabrication and alternatives selection

**Fig. 2:**
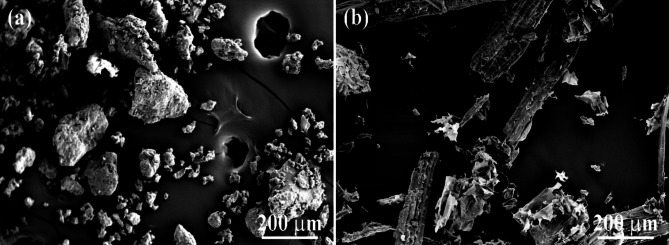
Scanning electron microscopic image of biomass fillers (**a**) Flax seed meal, (**b**) Rapeseed straw.

The formulations were developed using variants of PLA and agricultural by-products (rapeseed straw flax seed meal). PLA pellets (Ingeo 2003D) with 1.24 g/cm^3^ of specific gravity were bought from Nature Works (Minnetonka, MN, USA). Agricultural by-products used as fillers included rapeseed straw kindly provided by Mikó Stroh Borotai-Laska Ltd. in Hungary and flax seed meal acquired from a farmer in Himachal Pradesh, India. Both fillers were sieved in order to achieve particles of a uniform size (< 250 μm). Thereafter, they were rinsed with distilled water to eliminate any intrinsic contamination and subsequently dried at 80 °C until a stable mass was attained. Scanning electron microscopic (SEM) images of the agro-wastes are shown in Fig. 2.

**Fig. 3:**
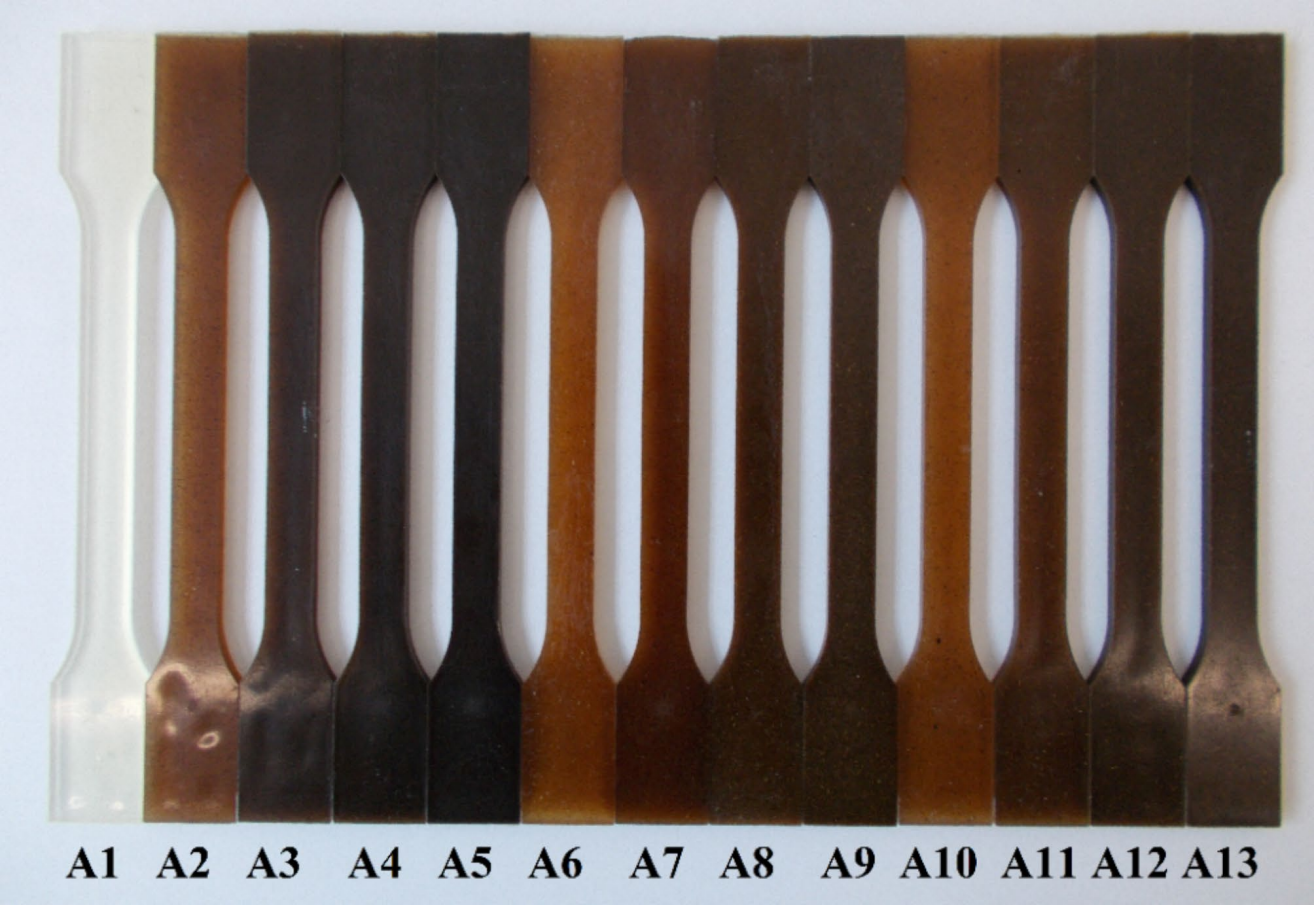
Photographic image of the fabricated sample alternatives.

Before melting, all components were dried at 80 °C for 4 h in a drying chamber (WGLL 125 BE, Huanghua, China). A co-rotating twin-screw extruder (model LTE 20–44, LabTech Engineering, Thailand) was used for processing. The temperature range for processing neat PLA and its biomass-filled composites was between 155 and 185 °C from the feeder to the die, with the screws rotating at a speed of 30 revolutions per min. A total of thirteen samples were prepared using this method, which included PLA and its composites with 2.5 wt% to 20 wt% bio-filler. The incorporated filler was either flax seed meal, rapeseed straw, or an equal combination of both. Table 1 summarizes compositional details of the produced biocomposite samples/alternatives. The extrudates underwent grinding with a grinder (model LZ120, LabTech Engineering, Thailand) to produce granulates that are suitable for subsequent processing into specimens for characterization. The specimens were fabricated using an injection molding machine (Allrounder 420 C-type) from Arburg, Germany. The process of injection molding was executed with an injection rate of 40 cm^3^/min, and a holding pressure sequence of 750-650-250 bar over a total duration of 15 s. The mold temperature was maintained at 30 °C, whereas the nozzle temperature was at 195 °C. Figure 3 presents the images of the fabricated biocomposite samples.

**Table 1:** Composition and designation of the fabricated sample alternatives.

Ingredients (wt.%)	A_1_	A_2_	A_3_	A_4_	A_5_	A_6_	A_7_	A_8_	A_9_	A_10_	A_11_	A_12_	A_13_
PLA	100	97.5	95	90	80	97.5	95	90	80	97.5	95	90	80
Flax seed meal	0	2.5	5	10	20	0	0	0	0	1.25	2.5	5	10
Rapeseed straw	0	0	0	0	0	2.5	5	10	20	1.25	2.5	5	10

### Characterizations and criteria selection

The tensile mechanical properties (Criterion 1 (C_1_): tensile strength; Criterion 2 (C_2_): Young’s modulus) were ascertained in accordance with the ISO 527 standard. The flexural mechanical properties (Criterion 3 (C_3_): flexural stress at conventional deflection; Criterion 4 (C_4_): flexural modulus) were determined in accordance with the ISO 178 standard. A 10 kN load cell was incorporated into an universal testing equipment (Instron 5582, Norwood, Massachusetts, USA), which was utilized to conduct both measurements. For the tensile tests 1 A type specimens were applied according to the ISO 527-2 standard. A tension rate of 5 mm/min was applied to a grasped length of 100 mm. Additionally, rectangular specimens measuring 4 mm x 10 mm with a 64 mm span length between the supports were used in the flexural tests, which were conducted at a crosshead speed of 5 mm/min. The results reported are the averages of five consecutive measurements in both cases. The Shore D hardness (Criterion 5) of the specimens was assessed using a digital hardness tester (Sauter HDD 100-1, Balingen, Germany). The mean hardness values were derived from the outcomes of seven successive measurements. Charpy tests were conducted in accordance with the ISO 179 standard using a Ceast 6545 impact testing instrument (Pianezza, Italy) to assess the impact strength (Criterion 6 (C6)) of the biocomposite specimens. For the impact tests, unnotched rectangular specimens were used with a size of 4 mm × 10 mm × 80 mm. The testing instrument was equipped with a 15 J impact hammer and the span length was set to 62 mm. The results reported are the averages of five consecutive measurements.

The composites’ water uptake/absorption (*M*_*t*_ – Criterion 7 (C_7_)) was evaluated over a span of 55 days by submerging specimens (10 mm × 10 mm × 4 mm) in distilled water and periodically recording their weight. Prior to the measurement, the samples underwent drying at a temperature of 80 °C. The calculation of the percentile increase in weight was performed using Eq. (15).

15 $${M_t}\left[ \% \right]=\frac{{{W_w} - {W_d}}}{{{W_d}}} \times 100$$

where *W*_*w*_ is the weight of the specimens after submerging them for 55 days and *W*_*d*_ is their dry weight measured before submersion.

The biodegradation of the samples (Criterion 8 (C_8_)) was examined according to the ISO 20200 standard through a laboratory-scale composting process. For this test, rectangular specimens (20 mm × 20 mm × 1 mm) were created at 180 °C using a hydraulic press (Model: LP-20B, LabTech Engineering, Thailand). Samples were buried in a reactor filled with compost and maintained at a 50% relative humidity and temperature of 58 °C for 28 days within an ACS DY110 climate chamber (Angelantoni Test Technologies, Perugia, Italy). After four weeks of composting the fragments of the degraded specimens were collected and sieved. Residues passing the 2 mm holes of the sieve were considered as degraded as per the standard, while the larger fragments were weighed to determine the remaining mass. The cost of the biocomposites (Criterion 9 (C_9_)) was determined based on the cost of materials utilized in composite production.

## Results and discussion

### Influence of rapeseed straw and flax seed meal on the evaluated criteria

#### Quasi-static mechanical properties

**Fig. 4:**
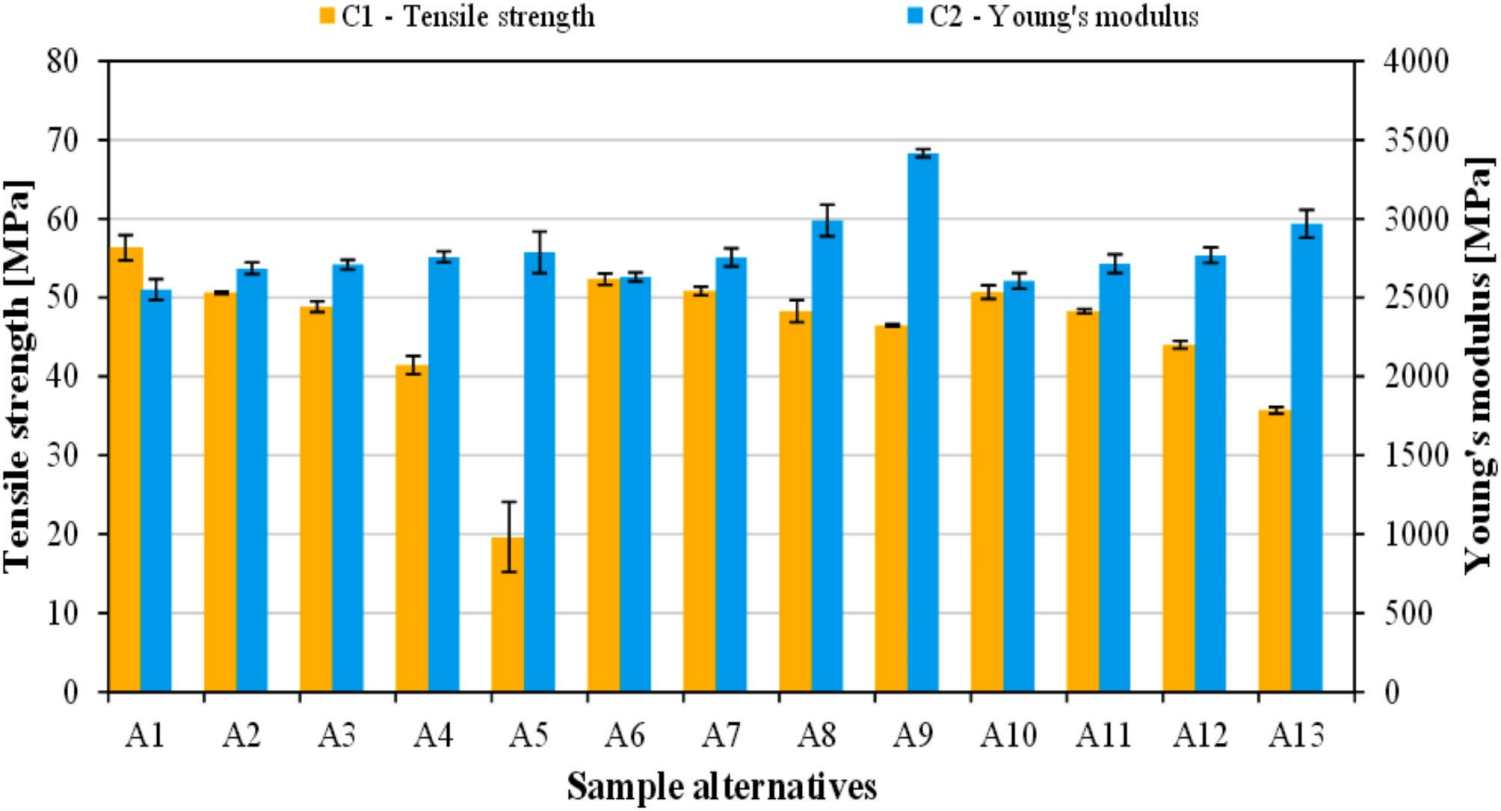
Results of C_1_ (tensile strength) and C_2_ (Young’s modulus).

The results of rapeseed straw and flax seed meal incorporation on C_1_ (tensile strength) and C_2_ (Young’s modulus) of the biocomposites is presented in Fig. 4. The tensile strength of unfilled PLA (sample A_1_) was found as highest (56.4 MPa) among the alternatives and it declined gradually with increasing bio-filler content. The rate of reduction greatly varied depending on the type of additive. Rapeseed straw-filled biocomposites only exhibited a slight loss in strength, retaining 46.5 MPa even at 20 wt% loading (sample A_9_). Meanwhile, when flax seed meal was used as biomass, the strength of PLA suffered a prominent drop, bottoming at 19.6 MPa (sample A_5_). The reason for rapeseed straw being superior is suggested to be due to its fibrous shape, unlike flax seed meal (see Fig. 1). Fiber-like shapes own larger specific surface areas compared to globular ones, which prominently increases the potential contact surface between the filler and the matrix, allowing improved strength properties. Similar behavior of NFRPCs filled with plant-based particles of various shapes was already reported in the literature^56^. Using both natural additives simultaneously resulted in interim strength values. According to the literature, biocomposites filled with natural fibers typically exhibit lower strength than the parent polymer, which is ascribed to the differences in polarity between the polymer matrix and the fillers due to which the interfacial adhesion is limited^57,58^.

On the other hand, Young’s modulus of PLA exhibited a prominent growth when the agricultural residues were incorporated into it. The modulus of neat PLA (A_1_– 2552 MPa) was outperformed by every composite alternative. Sample A_9_ with a rapeseed straw content of 20 wt% was found to be superior in this regard (3417 MPa), indicating that this type of agro-waste is highly effective in enhancing the stiffness of the polymer matrix. The improvement in Young’s modulus observed for the biomass-filled composites can be ascribed to the rigid nature of these fillers; their presence apparently hindered the molecular mobility of PLA chain molecules, thereby contributing to its stiffness. Based on the literature, it can also be assumed that Young’s modulus was able to increase even at decreasing tensile strength since it is less affected by the interphase between the components; the individual features of the components are more important in this respect. Similar observations were made by Robledo-Ortíz et al.^59^ for sugarcane straw-reinforced PLA composites.

**Fig. 5:**
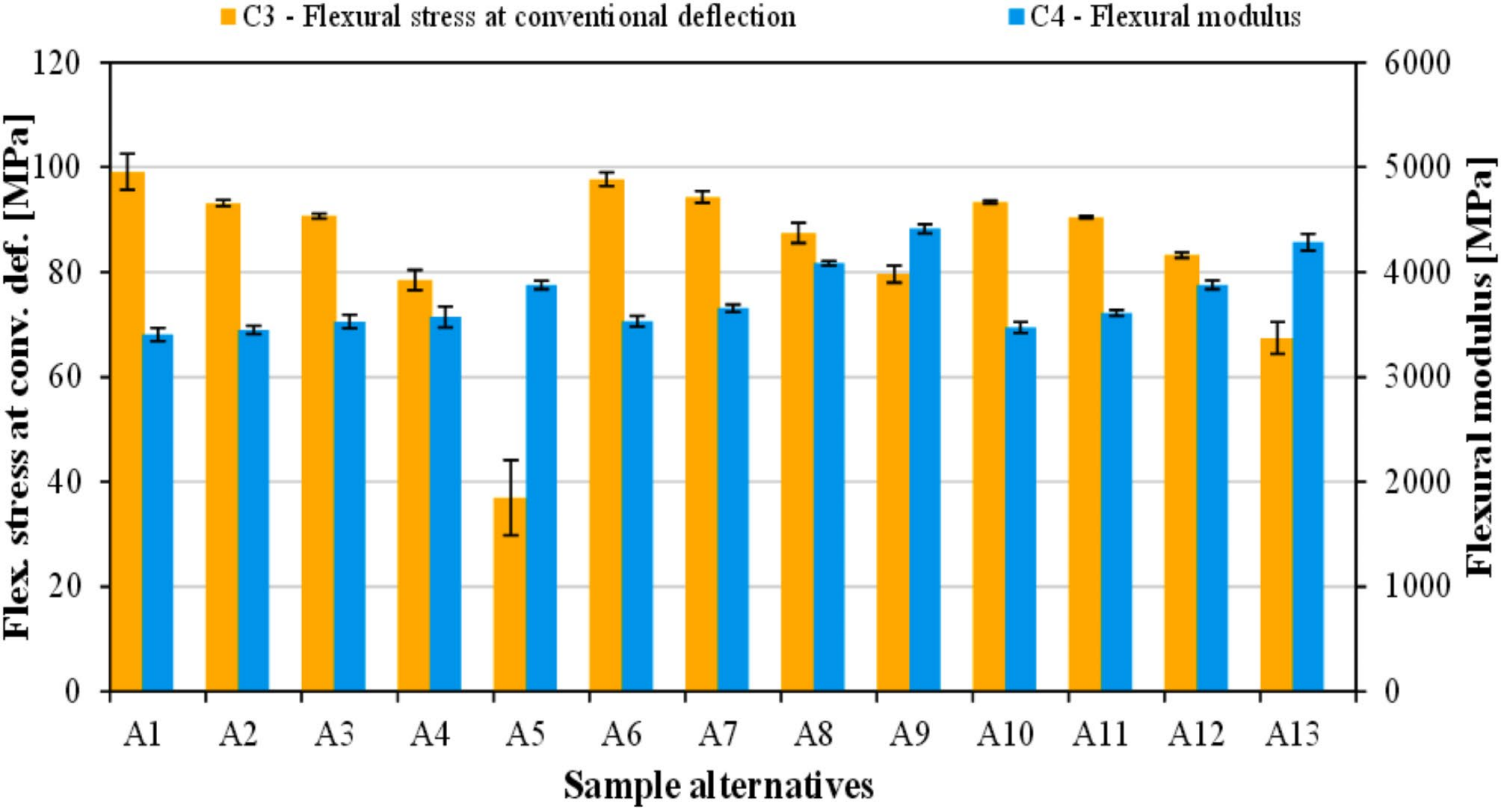
Results of C_3_ (flexural stress at conventional deflection), and C_4_ (flexural modulus).

Figure 5 presents the mechanical properties determined through 3-point bending tests, namely C_3_ (flexural stress at conventional deflection), and C_4_ (flexural modulus). The largest (99.1 MPa) and the smallest (36.9 MPa) C_3_ parameters were exhibited by unfilled PLA (A_1_) and 20 wt% flax seed meal containing biocomposite (A_5_), respectively.

Overall, the flexural stress at conventional deflection of rapeseed straw-filled biocomposites was close to that of neat PLA, suggesting that rapeseed straw has great potential as filler/reinforcement in polymer biocomposites. Meanwhile, increasing the weight% of flax seed meal in the biocomposites resulted in a considerable drop in flexural load-bearing capacity. Such is the case in many composites containing natural fillers as it is well described in the literature^60^. Contrary to the strength property, the flexural modulus of the biocomposites grew with an increased flax seed meal and rapeseed straw loading, which is in good agreement with the tensile test results. The lowest flexural modulus (3404 MPa) was exhibited by sample A_1_ (unfilled PLA), while the largest value (4415 MPa) was recorded for 20 wt% rapeseed straw added biocomposite (A_9_). Flax seed meal also improved the flexural modulus, however to a lesser extent. The composites containing 20 wt% (i) rapeseed straw; (ii) flax seed meal; (iii) flax seed meal/rapeseed straw increased the flexural modulus relatively by 29.7%, 25.8%, and 13.9%, respectively. Kuciel et al.^61^ reported similar results, namely decreased flexural strength but increased flexural modulus for PLA biocomposites filled with wood fibers.

#### Hardness and impact strength results

**Fig. 6:**
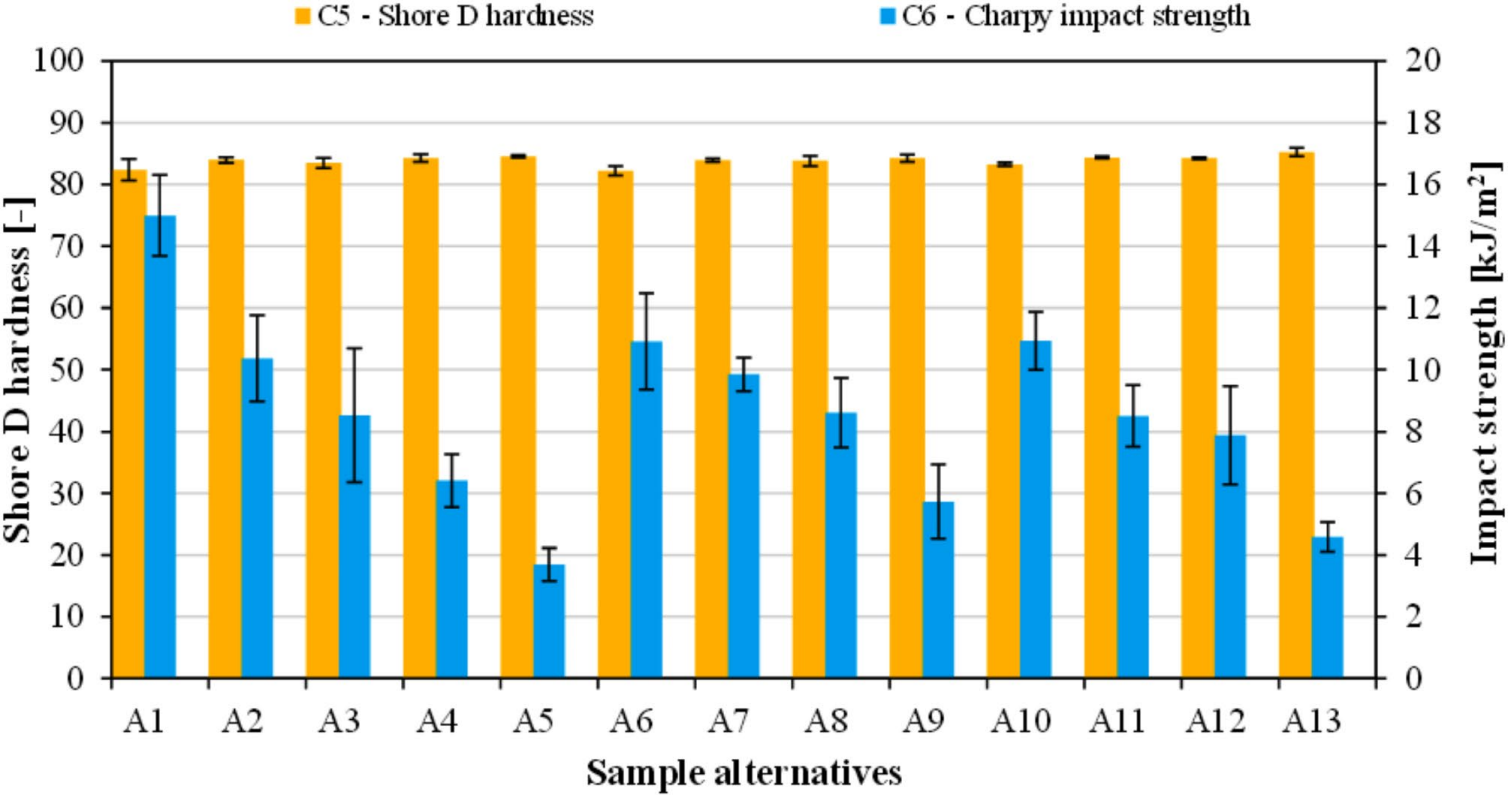
Results of C_5_ (Shore D hardness) and C_6_ (Charpy impact strength).

Figure 6 shows the influence of rapeseed straw and flax seed meal on the C_5_ (Shore D hardness) and C_6_ (Charpy impact strength) of the fabricated samples. The Shore D hardness values fluctuated in the small range of 82.3 and 85.2 where PLA was found to own the lowest resistance against the indenter. With growing filler content the hardness of all biocomposites increased analogously. The noted increase in hardness values corresponding to higher lignocellulosic content aligns well with existing literature^62,63^. It is suggested that both agro-wastes improve the resistance of the polymer’s surface to deform plastically. In this respect, there were no marked differences between the applied biomass types. The relatively low changes in hardness can be explained by the fact that PLA itself is a rather rigid plastic showing little to no plastic deformations. Contrarily, the toughness of PLA (A_1_ – 15 kJ/m^2^) dropped drastically in the presence of even a small amount of flax seed meal or rapeseed straw. The biocomposites containing 2.5 wt% biomass of various types exhibited an impact strength of 10.4 kJ/m^2^ (A_2_), 10.9 kJ/m^2^ (A_6_), and 10.9 kJ/m^2^ (A_10_). Among the biocomposites with maximum filler loading the PLA/rapeseed straw (A_9_) owned the highest impact strength of 5.7 kJ/m^2^, while PLA/flax seed meal (A_5_) was the lowest (3.7 kJ/m^2^). Such reduction in toughness is often reported in the literature for natural fiber filled biocomposites and is often attributed to the limited interfacial adhesion between the polymer matrix and the filler particles. For instance, Andrzejewski et al.^30^ reported a drop in impact strength from 4.5 kJ/m^2^ to around 2 kJ/m^2^ at 10 wt% cork/wood composites.

#### Water uptake, biodegradation and cost of the biocomposites

**Fig. 7:**
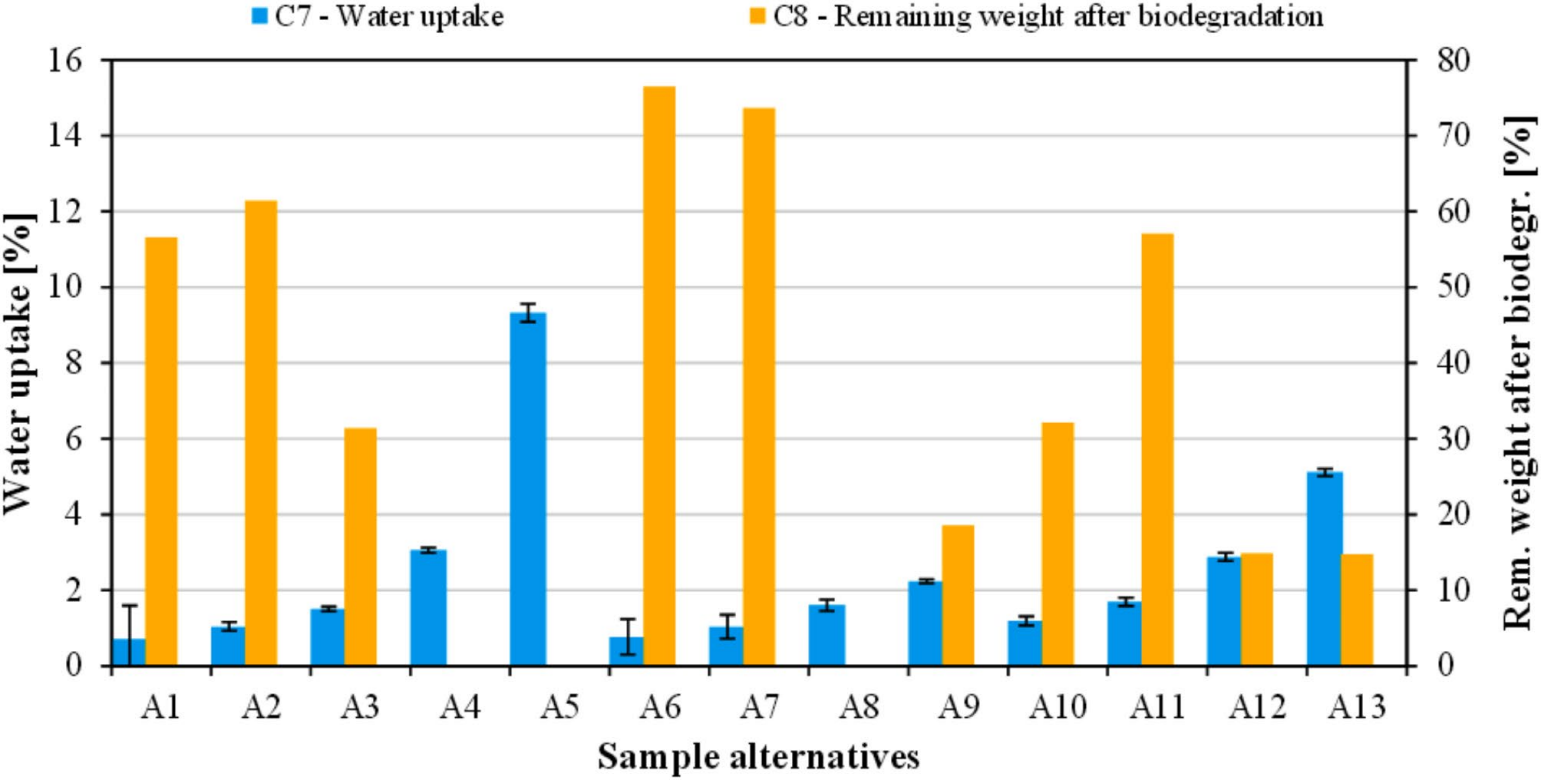
Results of C_7_ (water uptake) and C_8_ (remaining weight after biodegradation).

Figure 7 illustrates the influence of rapeseed straw and flax seed meal on C_7_ (water uptake) and C_8_ (remaining weight after biodegradation) of the investigated biocomposites. Neat PLA (A_1_) exhibited the lowest water absorption (0.71%), which can be ascribed to the hydrophobic nature of this polymer^64^. Natural particles, including flax seed meal and rapeseed straw are composed of cellulose, hemicellulose, and lignin. Since all these substances possess a large amount of hydroxyl groups on their surface, they are rather hydrophilic and tend to absorb a relatively high amount of moisture. Additionally, natural fibers are rather cellular, which also contributes to their capability to absorb water. The fillers’ hydrophilic characteristics and their evident incompatibility with the PLA matrix facilitated water transport, resulting in biocomposite alternatives becoming increasingly prone to water absorption as biomass content rises. The literature has reported similar findings regarding increased water absorption with higher concentrations of natural fiber^65^. Based on the results, PLA/flax seed meal biocomposites absorbed the largest amount of water by far (up to 9.3%), while the water absorption of PLA/rapeseed straw biocomposites peaked at 2.2%. This is a further indicator of PLA and flax seed meal being rather incompatible and aligns with the results of mechanical tests.

**Fig. 8:**
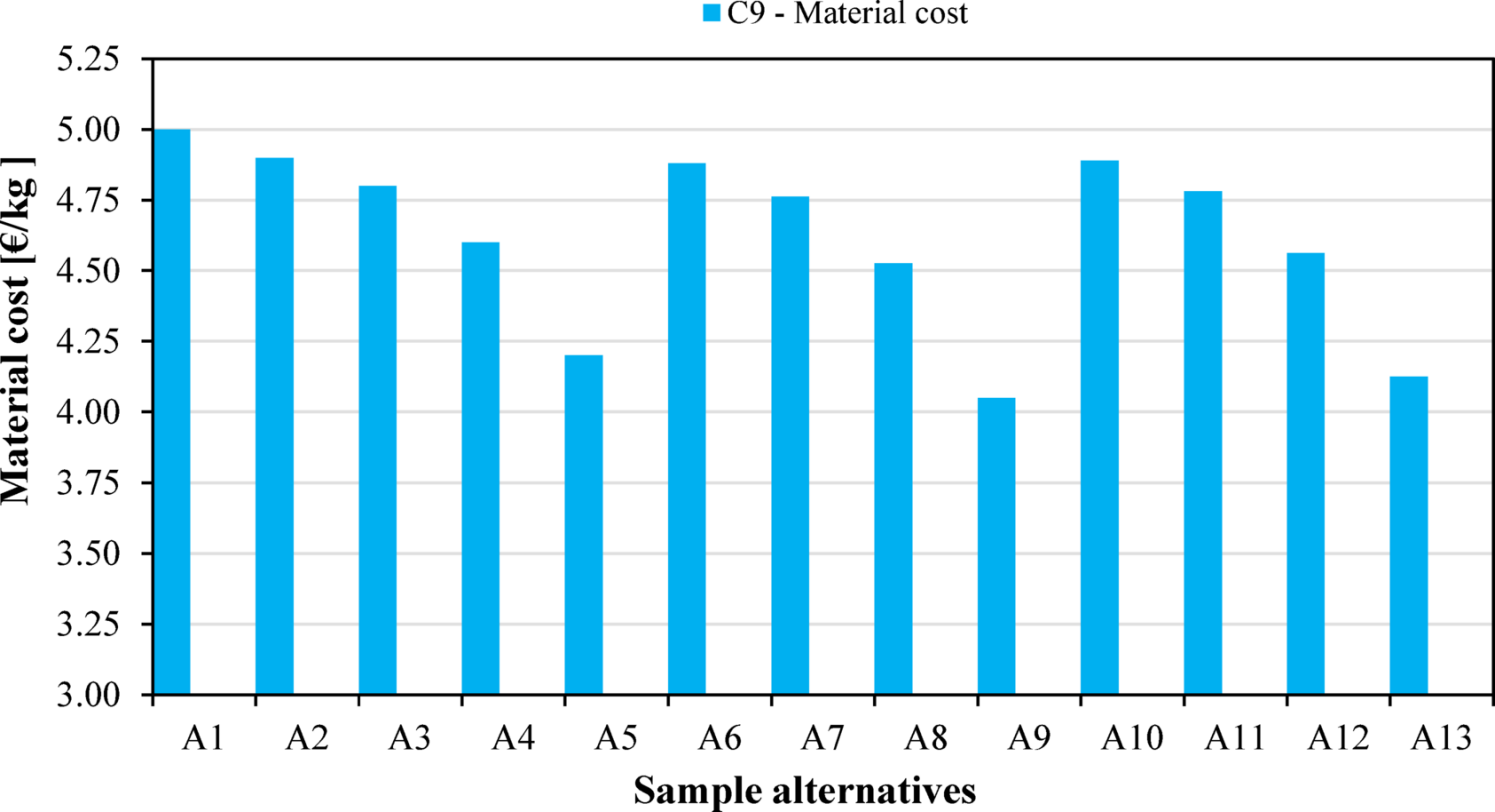
Cost analysis (C_9_) of the biocomposites.

The remaining weight of the samples after degradation in controlled compost soil is shown in Fig. 7. Disintegration of the samples was followed for a total of 28 days. Unfilled PLA (A_1_) had a remaining weight of 56.6% after soil burial composting. Along with that, samples containing low amounts of natural fillers proved to be the most resistant to biodegradation. On the other hand, biocomposites owning high agro-waste content disintegrated to a great extent by the end of the test. Flax seed meal was determined to be the most effective addition in this regard; at the conclusion of the fourth week, both alternative A_4_ and alternative A_5_ (PLA filled with 10 wt% and 20 wt% flax seed meal, respectively) had decomposed completely. The other two biocomposites containing 20 wt% fillers (A_9_ and A_13_) also underwent a major disintegration, having a remaining weight of 18.6% and 14.7%, respectively. These results also suggest that flax seed meal facilitates biodegradation more effectively than rapeseed straw does. The material cost (C_9_; €/kg) of the fabricated samples was calculated and presented in Fig. 8. The calculations were based on the cost of PLA (5 €/kg), rapeseed straw (0.25 €/kg), and flax seed meal (1 €/kg). The cost of unfilled PLA (i.e., alternative A_1_) is highest (5 €/kg). The cost was found to decrease with increased agricultural byproduct loadings and remained lowest for alternative A_9_ having 20 wt% rapeseed straw content.

### Ranking of the biocomposites

The biocomposites were ranked based on their assessed physical and mechanical qualities. The selection criteria included the evaluated properties: tensile strength – MPa (C_1_; higher-the-better), Young’s modulus – MPa (C_2_; higher-the-better), flexural stress at conventional deflection – MPa(C_3_; higher-the-better), flexural modulus – MPa (C_4_; higher-the-better), Shore D hardness – (C_5_; higher-the-better), impact strength – kJ/m^2^ (C_6_; higher-the-better), water uptake – % (C_7_; lower-the-better), remaining weight after biodegradation – % (C_8_; lower-the-better) and material cost– €/kg (C_9_; lower-the-better). The best biocomposite was selected using the combined CRITIC-CoCoSo technique, which included all assessed parameters. The determination of the weights of the criteria was carried out using the CRITIC approach, as explained in the “Optimization methodology” section. A decision matrix was developed for the specified alternative and criterion using Eq. 1 and given in Table 2.

**Table 2:** The formulated decision matrix.

Samples	C_1_	C_2_	C_3_	C_4_	C_5_	C_6_	C_7_	C_8_	C_9_
A_1_	56.36	2552.04	99.09	3404.32	82.34	14.99	0.71	56.56	5.00
A_2_	50.64	2685.68	93.13	3447.96	83.95	10.37	1.04	61.37	4.90
A_3_	48.87	2711.16	90.7	3527.50	83.45	8.53	1.50	31.37	4.80
A_4_	41.46	2758.38	78.47	3573.23	84.28	6.41	3.05	0.00	4.60
A_5_	19.63	2788.77	36.95	3877.08	84.53	3.69	9.32	0.00	4.20
A_6_	52.35	2631.13	97.72	3530.17	82.25	10.92	0.77	76.49	4.88
A_7_	50.87	2755.49	94.34	3654.56	83.90	9.85	1.04	73.61	4.76
A_8_	48.29	2989.42	87.47	4083.65	83.80	8.61	1.60	0.00	4.53
A_9_	46.50	3416.38	79.65	4414.6	84.23	5.73	2.23	18.57	4.05
A_10_	50.69	2606.83	93.36	3471.55	83.28	10.93	1.19	32.11	4.89
A_11_	48.27	2716.20	90.49	3611.93	84.35	8.50	1.69	57.11	4.78
A_12_	44.03	2769.58	83.24	3880.03	84.23	7.88	2.88	14.83	4.56
A_13_	35.74	2968.50	67.45	4283.94	85.23	4.58	5.11	14.73	4.13

**Fig. 9:**
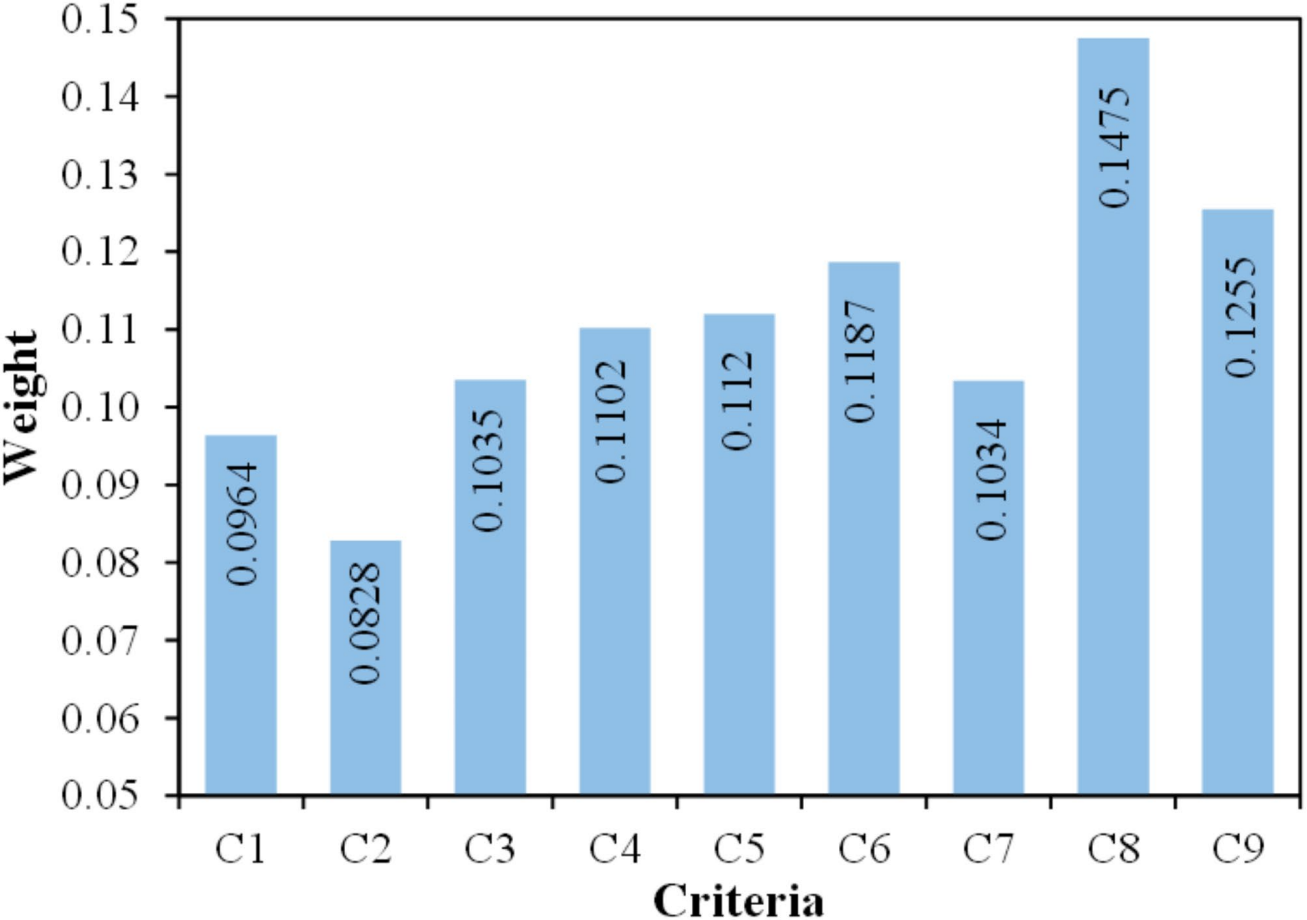
Results of criteria weight.

The CRITIC approach included normalizing the decision matrix using Eqs. 2 and 3, and the results are shown in Table 3. Thereafter, the index value for each criterion was determined using Eqs. 6 and 7. The index values of various criteria are C_1_ = 2.0168, C_2_ = 1.7319, C_3_ = 2.167, C_4_ = 2.3067, C_5_ = 2.3448, C_6_ = 2.4853, C_7_ = 2.1638, C_8_ = 3.0862, and C_9_ = 2.6273. Finally, the weight of each criterion was computed using Eq. 8 and depicted in Fig. 9.

**Table 3:** The normalized decision matrix.

Samples	C_1_	C_2_	C_3_	C_4_	C_5_	C_6_	C_7_	C_8_	C_9_
A_1_	1.0000	0.0000	1.0000	0.0000	0.0302	1.0000	1.0000	0.2606	0.0000
A_2_	0.8443	0.1546	0.9041	0.0432	0.5705	0.5912	0.9617	0.1977	0.1053
A_3_	0.7961	0.1841	0.8650	0.1219	0.4027	0.4283	0.9082	0.5899	0.2105
A_4_	0.5943	0.2387	0.6682	0.1672	0.6812	0.2407	0.7282	1.0000	0.4211
A_5_	0.0000	0.2739	0.0000	0.4679	0.7651	0.0000	0.0000	1.0000	0.8421
A_6_	0.8908	0.0915	0.9780	0.1246	0.0000	0.6398	0.9930	0.0000	0.1263
A_7_	0.8505	0.2354	0.9236	0.2477	0.5537	0.5451	0.9617	0.0377	0.2526
A_8_	0.7803	0.5060	0.8130	0.6724	0.5201	0.4354	0.8966	1.0000	0.4947
A_9_	0.7316	1.0000	0.6872	1.0000	0.6644	0.1805	0.8235	0.7572	1.0000
A_10_	0.8456	0.0634	0.9078	0.0665	0.3456	0.6407	0.9443	0.5802	0.1158
A_11_	0.7797	0.1899	0.8616	0.2055	0.7047	0.4257	0.8862	0.2534	0.2316
A_12_	0.6643	0.2517	0.7449	0.4709	0.6644	0.3708	0.7480	0.8061	0.4632
A_13_	0.4386	0.4818	0.4908	0.8707	1.0000	0.0788	0.4890	0.8074	0.9158

According to the weights assigned to the criterion in Fig. 9, biodegradation (C_8_) has the highest priority among the others, with a weight of 0.1475. The criterion of cost (C_9_) has the second highest importance, with a weight of 0.1255. The criterion of impact strength (C_6_) has the third highest level of significance, with a weight of 0.1187. The criterion of hardness (C_5_) and flexural modulus (C_4_) are ranked as the fourth and fifth priorities, with similar scores of around 0.1120 and 0.1102, respectively. The criterion of flexural strength (C_3_) and water uptake (C_7_) are ranked as the sixth and seventh priorities, with similar scores of around 0.1035 and 0.1034, respectively. The criterion of tensile strength (C_1_) and tensile modulus (C_2_) have weights of 0.0964 and 0.0828, respectively, making them the second-lowest and lowest important criteria.

**Fig. 10:**
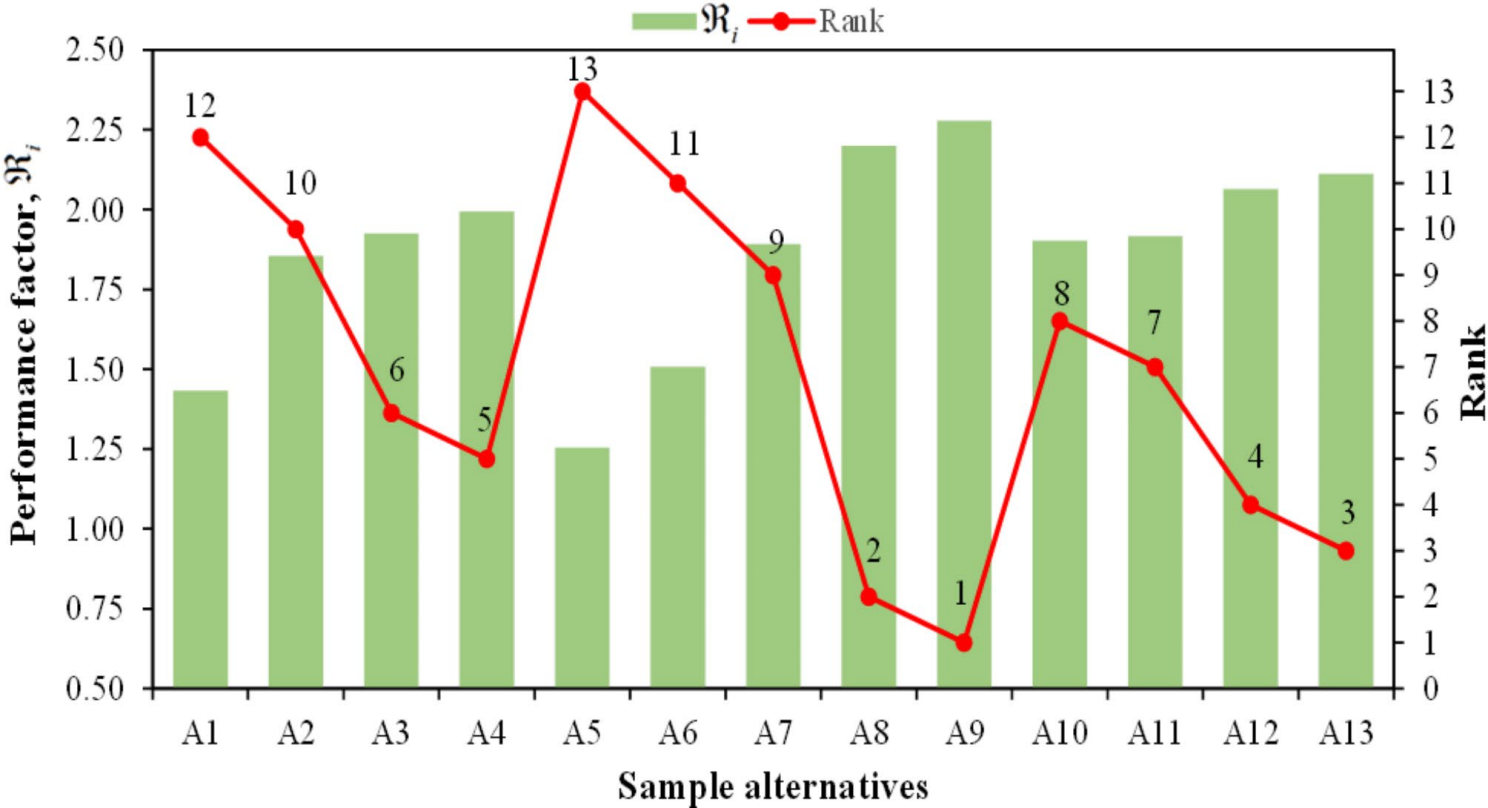
Performance factor (${\Re _i}$) and final rank of the alternatives.

Next, the biocomposite alternatives were ranked using the CoCoSo method with respect to the defined criteria, detailed in the “Optimization methodology” section. After the decision matrix normalization, the weighted sum comparability score (${\varpi _{ij}}$) and the weighted power comparability score ($\varpi _{{ij}}^{ * }$) for each alternative were determined using Eqs. 9 and 10. The results of ${\varpi _{ij}}$and$\varpi _{{ij}}^{ * }$ are listed in Table 4 for each alternative. Following this step, three appraisal scores${\Re _{ia}}$, ${\Re _{ib}}$, and ${\Re _{ic}}$were calculated using the relation presented in Eqs. 11–13 and listed in Table 4. In the last step, the performance factor (${\Re _i}$) for each alternative was determined using Eq. 14 which indicates their rank. The rank of the biocomposite alternatives along with ${\Re _i}$ value is depicted in Fig. 10. Results obtained from the proposed integrated CRITIC-CoCoSo decision-making approach show that alternative A_9_, with the highest performance score (2.2779), is the most promising and top alternative among other options. With a performance score of 2.2003 and 2.1110, alternative A_9_ and alternative A_13_ are second and third-ranked alternatives, respectively. As represented in Table 4; Fig. 10, alternative A_5_ is ranked as the worst alternative among the evaluated biocomposites by getting the lowest performance score of 1.2543. It can be observed that among these thirteen biocomposite alternatives, 80 wt% PLA + 20 wt% rapeseed straw is the best alternative, while 80 wt% PLA + 20 wt% flax seed meal is the worst.

**Table 4:** Results of CoCoSo method.

Samples	${\varpi _{ij}}$	$\varpi _{{ij}}^{ * }$	${\Re _{ia}}$	${\Re _{ib}}$	${\Re _{ic}}$	${\Re _i}$
A_1_	0.4638	5.4958	0.0561	2.3041	0.6342	1.4325
A_2_	0.4684	7.9533	0.0793	2.8310	0.8963	1.8549
A_3_	0.4982	8.1706	0.0816	2.9506	0.9226	1.9239
A_4_	0.5451	8.2867	0.0831	3.0914	0.9399	1.9942
A_5_	0.4131	4.7672	0.0488	2.0254	0.5513	1.2543
A_6_	0.4029	6.3208	0.0633	2.3259	0.7156	1.5072
A_7_	0.4878	8.0412	0.0803	2.8975	0.9077	1.8907
A_8_	0.6876	8.5972	0.0874	3.5102	0.9881	2.2003
A_9_	0.7528	8.6435	0.0884	3.6818	1.0000	2.2779
A_10_	0.5006	8.0279	0.0803	2.9265	0.9076	1.9023
A_11_	0.4902	8.1742	0.0815	2.9315	0.9221	1.9157
A_12_	0.5867	8.4349	0.0849	3.2256	0.9601	2.0642
A_13_	0.6349	8.4050	0.0851	3.3389	0.9621	2.1110

### Sensitivity analysis and validation of the proposed MCDM model

#### Sensitivity analysis

**Fig. 11:**
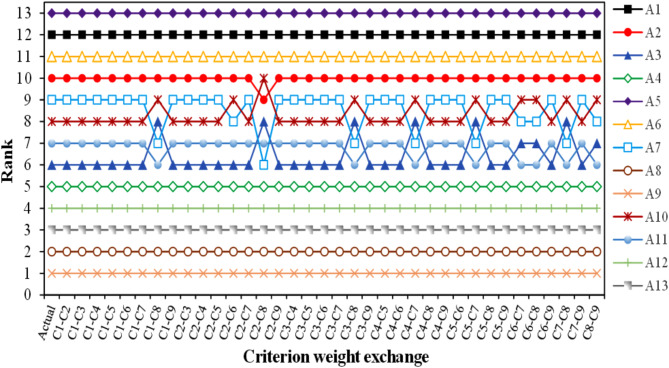
Rank sensitivity analysis.

A sensitivity study has been conducted to assess the stability of the combined CRITIC-CoCoSo approach and verify the obtained findings. A sensitivity study was performed on the CRITIC-CoCoSo models by creating additional weights and analyzing their impact on the ranks of the alternatives. The criterion weights were recalibrated using the exchanged criteria weights, resulting in new vectors of weights. There are a total of nine criteria (C_1_-C_9_); resulting in a total of thirty-six potential interchanges. Figure 11 presents the thirty-six sets of rankings for different biocomposite choices. Upon analyzing Fig. 11, it can be inferred that the ranks of the alternatives were not considerably impacted by the interchange of criterion weights. Throughout all weight exchange situations, alternative A_9_ consistently maintains its superiority, whereas alternative A_5_ consistently stays in the lowest position. In some weight exchange conditions, alternatives rated sixth to tenth are seen to switch ranks. Nevertheless, these modifications have little impact on the ultimate outcomes of the model, as seen by the Spearman correlation coefficient value above 0.95 in all weight exchange situations. This demonstrates the resilience of the combined CRITIC-CoCoSo approach in choosing the optimal candidate from a range of accessible composites.

#### Comparative analysis with other MCDM models

**Fig. 12:**
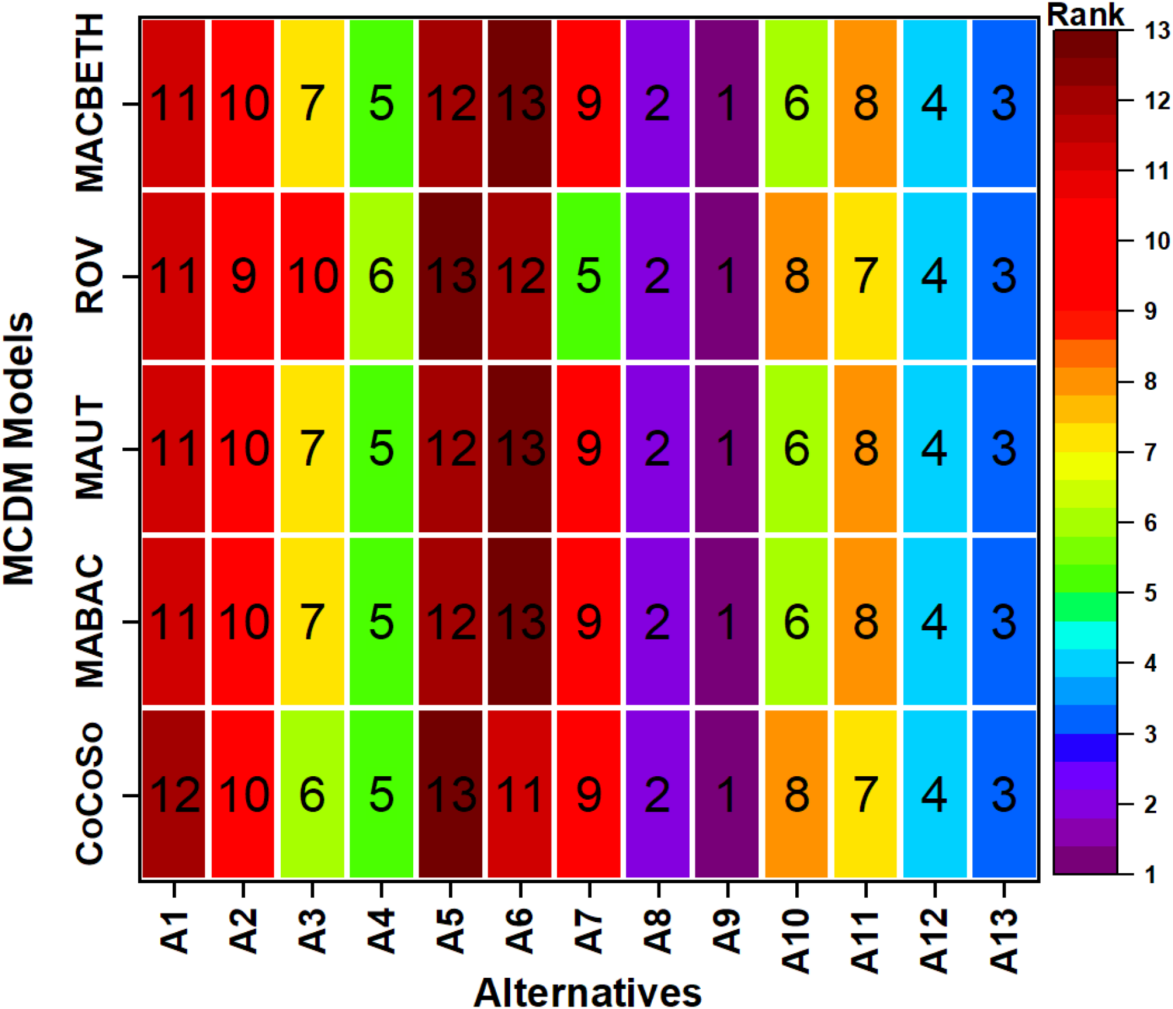
Rank validation analysis.

For the purpose of assessing the effectiveness of the suggested technique and the accuracy of the CoCoSo computations, four distinct MCDM models, namely MABAC, MAUT, ROV, and MACBETH were used. These models were utilized to evaluate and prioritize the alternatives based on the predefined criteria in this research and results are presented in Fig. 12. As seen in Fig. 12, all the MCDM techniques used to validate the accuracy of the final ranking derived from the CoCoSo approach consistently endorse the final ranking. The comparison of the models revealed that alternative A_9_ is the top-ranked option, with only minor variations in ranking seen following alternative A_4_. The discrepancies in the rankings may originate from variations in the computation and normalization methodologies used in various applicable MCDM systems. The CoCoSo approach has a strong association with the MABAC, MAUT, ROV, and MACBETH methods, as determined by the calculation of the Spearman correlation coefficient. The correlation coefficients between the rank of the CoCoSo method and the MABAC, MAUT, and MACBETH techniques remain greater than 0.97 while it was 0.90 for ROV. The alternative A_9_ is consistently chosen as the optimal choice in all ways, indicating that the suggested technique is validated based on these findings.

## Conclusions

Utilizing waste to produce polymeric composites offers cost-effective, sustainable alternative materials for various structural uses. Hence, thirteen distinct biocomposites are produced by including flax seed meal and rapeseed straw as reinforcing materials, varying weight percentages ranging from 0 to 20%, into poly(lactic acid). The findings of the study can be summarized as follows:

- Adding higher amounts of flax seed meal/rapeseed straw to poly(lactic acid) led to improvements in the hardness, Young’s modulus, water absorption, biodegradability, and flexural modulus of the biocomposites. In contrast, the poly(lactic acid) biocomposites’ cost, tensile, flexural, and impact strengths declined as the flax seed meal/rapeseed loading increased.
- The suggested integrated CRITIC-CoCoSo approach has determined the biocomposite material consisting of 20 wt% rapeseed straw and 80 wt% poly(lactic acid) as the most suited option based on the stated criteria and alternatives, which exhibited a tensile strength of 46.5 MPa, a flexural strength of 79.6 MPa, Young’s modulus of 3.41 GPa, a flexural modulus of 4.41 GPa, impact strength of 5.7 kJ/m^2^, a Shore D hardness of 84.2, and the remaining mass after the composting was 18%.
- Sensitivity analysis and comparison with the other MCDM models, such as CRITIC-MABAC, CRITIC-MAUT, CRITIC-ROV, and CRITIC-MACBETH, evaluate the coherence and resilience of the solutions. The Spearman’s rank correlation coefficients, which exceed 0.9, demonstrate the stability of the ranks across various decision-making procedures in connection to weight sensitivity and the suggested method.

The research may be expanded by examining the elevated concentration of agricultural by-products in various polymers. Future investigations may focus on the remaining properties, including dynamic mechanical properties, chemical properties, and life cycle analysis. Furthermore, this study excludes the uncertainty in decision-making regarding selecting biocomposite materials, an area for future investigation. This research assists designers and manufacturers in selecting materials for sustainable composites. The findings and analyses of the study can be advantageous to academics and scientists engaged in the field of sustainability.

## Data Availability

The data is available from the corresponding author upon request.

## References

[1] Taha, Z. T., Major, A. Á. & Ronkay, F. Effect of reprocessing on the crystallization of different polyesters. *Acta Tech. Jaurinensis* **17**, 1–7 (2024).

[2] Buruiana, D. L., Georgescu, P. L., Carp, G. B. & Ghisman, V. Recycling micro polypropylene in modified hot asphalt mixture. *Sci. Rep.* **13**, 3639 (2023). 36871062 10.1038/s41598-023-30857-9 PMC9985627

[3] Zeng, W. et al. A general strategy for recycling polyester wastes into carboxylic acids and hydrocarbons. *Nat. Commun.* **15**, 160 (2024). 38167384 10.1038/s41467-023-44604-1 PMC10761813

[4] Jurado-Contreras, S., Navas-Martos, F. J., García-Ruiz, Á., Rodríguez-Liébana, J. A. & Rubia, M. D. L. Obtaining cellulose nanocrystals from Olive tree pruning waste and evaluation of their influence as a reinforcement on biocomposites. *Polymers* **15**, 4251 (2023). 37959931 10.3390/polym15214251 PMC10647253

[5] Vanderfleet, O. M. & Cranston, E. D. Production routes to tailor the performance of cellulose nanocrystals. *Nat. Rev. Mater.* **6**, 124–144 (2021).

[6] Xia, Q. et al. A strong, biodegradable and recyclable lignocellulosic bioplastic. *Nat. Sustain.* **4**, 627–635 (2021).

[7] Yaradoddi, J. S. et al. Biodegradable carboxymethyl cellulose based material for sustainable packaging application. *Sci. Rep.* **10**, 21960 (2020). 33319818 10.1038/s41598-020-78912-z PMC7738677

[8] Rosenboom, J. G., Langer, R. & Traverso, G. Bioplastics for a circular economy. *Nat. Rev. Mater.* **7**, 117–137 (2022). 35075395 10.1038/s41578-021-00407-8 PMC8771173

[9] Wang, L., Tong, Z., Ingram, L. O., Cheng, Q. & Matthews, S. Green composites of Poly (lactic acid) and sugarcane Bagasse residues from bio-refinery processes. *J. Polym. Environ.* **21**, 780–788 (2013).

[10] Lokesh, K. S. et al. Mechanical characterization & regression analysis of Calamus rotang based hybrid natural fibre composite with findings reported on retrieval bending strength. *Sci. Rep.* **14**, 3943 (2024). 38365832 10.1038/s41598-024-53570-7 PMC10873315

[11] Barba, B. J. D., Seko, N., Madrid, J. F. & Penaloza, D. P. Jr. Modified Abaca fiber prepared by radiation-induced graft polymerization as a reinforcement for unsaturated polyester resin composites. *Polym. J.* **56**, 97–105 (2024).

[12] VarunKumar, T. et al. An examining the static and dynamic mechanical characteristics of milled Ramie root reinforced polyester composites. *Sci. Rep.* **13**, 17054 (2023). 37816872 10.1038/s41598-023-44088-5 PMC10564739

[13] Rakowska, J., Węgrzyn, M. & Rudnik, E. Impact of ionic liquids on absorption behaviour of natural fibers/biopolyethylene biocomposites. *Sci. Rep.* **11**, 20483 (2021). 34650169 10.1038/s41598-021-99956-9 PMC8516865

[14] Chomachayi, M. D., Blanchet, P., Hussain, A. & Pepin, S. Development of a novel sandwich-structured composite from biopolymers and cellulose microfibers for Building envelope applications. *Sci. Rep.* **13**, 21955 (2023). 38082144 10.1038/s41598-023-49273-0 PMC10713545

[15] Rusin-Żurek, K. & Kuciel, S. Strength properties and ability to dissipate mechanical energy of biopolypropylene basalt/cellulose composites with the addition of antibacterial turmeric. *Sci. Rep.* **14**, 820 (2024). 38191797 10.1038/s41598-023-51145-6 PMC10774429

[16] Lendvai, L. & Patnaik, A. The effect of coupling agent on the mechanical properties of injection molded polypropylene/wheat straw composites. *Acta Tech. Jaurinensis* **15**, 232–238 (2022).

[17] Lendvai, L. Lignocellulosic agro-residue/polylactic acid (PLA) biocomposites: Rapeseed straw as a sustainable filler. *Clean. Mater.* **9**, 100196 (2023).

[18] Kulkarni, N. D., Saha, A. & Kumari, P. The development of a low-cost, sustainable bamboo-based flexible bio composite for impact sensing and mechanical energy harvesting applications. *J. Appl. Polym. Sci.* **140**, e54040 (2023).

[19] Saha, A., Kumar, S. & Kumar, A. Influence of pineapple leaf particulate on mechanical, thermal and biodegradation characteristics of pineapple leaf fiber reinforced polymer composite. *J. Polym. Res.* **28**, 66 (2021).

[20] Kumar, S., Saha, A. & Zindani, D. Agro-waste-based polymeric composite laminates for aerospace cabin interior and identification of their optimal configuration. *Biomass Convers. Biorefinery*. 10.1007/s13399-023-04914-2 (2023).

[21] Kumar, S. & Saha, A. Utilization of coconut shell biomass residue to develop sustainable biocomposites and characterize the physical, mechanical, thermal, and water absorption properties. *Biomass Convers. Biorefinery*. **14**, 12815–12831 (2024).

[22] Kumar, S. & Saha, A. Effects of particle size on structural, physical, mechanical and tribology behaviour of agricultural waste (corncob micro/nano-filler) based epoxy biocomposites. *J. Mater. Cycles Waste Manag.* **24**, 2527–2544 (2022).

[23] Shahdan, D., Rosli, N. A., Chen, R. S., Ahmad, S. & Gan, S. Strategies for strengthening toughened poly(lactic acid) blend via natural reinforcement with enhanced biodegradability: A review. *Int. J. Biol. Macromol.* **251**, 126214 (2023). 37572810 10.1016/j.ijbiomac.2023.126214

[24] Liao, Z. F. et al. Preparation and characterization of PLA/rice straw fiber composite. *Appl. Mech. Mater.* **71–78**, 1154–1157. (2011).

[25] Yussuf, A. A., Massoumi, I. & Hassan, A. Comparison of polylactic Acid/kenaf and polylactic acid/rise husk composites: The influence of the natural fibers on the mechanical, thermal and biodegradability properties. *J. Polym. Environ.* **18**, 422–429 (2010).

[26] Finkenstadt, V. L. et al. Poly(lactic acid) green composites using oilseed coproducts as fillers. *Ind. Crops Prod.* **26**, 36–43 (2007).

[27] Kariz, M., Sernek, M., Obućina, M. & Kuzman, M. K. Effect of wood content in FDM filament on properties of 3D printed parts. *Mater. Today Commun.* **14**, 135–140 (2018).

[28] Scaffaro, R., Maio, A., Gulino, E. F., Alaimo, G. & Morreale, M. Green composites based on PLA and agricultural or marine waste prepared by FDM. *Polymers* **13**, 1361 (2021). 33919389 10.3390/polym13091361 PMC8122657

[29] Scaffaro, R., Maio, A., Gulino, E. F. & Megna, B. Structure-property relationship of PLA-Opuntia Ficus indica biocomposites. *Compos. Part. B Eng.* **167**, 199–206 (2019).

[30] Andrzejewski, J., Szostak, M., Barczewski, M. & Łuczak, P. Cork-wood hybrid filler system for polypropylene and poly(lactic acid) based injection molded composites. Structure evaluation and mechanical performance. *Compos. Part. B Eng.* **163**, 655–668 (2019).

[31] Huang, C. C., Chang, C. W., Jahan, K., Wu, T. M. & Shih, Y. F. Effects of the grapevine biochar on the properties of PLA composites. *Materials* **16**, 816 (2023). 36676553 10.3390/ma16020816 PMC9867296

[32] Saleh, N., Gaber, M. N., Eldosoky, M. A. & Soliman, A. M. Vendor evaluation platform for acquisition of medical equipment based on multi-criteria decision-making approach. *Sci. Rep.* **13**, 12746 (2023). 37550351 10.1038/s41598-023-38902-3 PMC10406946

[33] Ashraf, S. et al. A model for emergency supply management under extended EDAS method and spherical hesitant fuzzy soft aggregation information. *Sci. Rep.* **13**, 8375 (2023). 37225781 10.1038/s41598-023-35390-3 PMC10209168

[34] Beheshtinia, M. A., Bahrami, F., Fathi, M. & Asadi, S. Evaluating and prioritizing the healthcare waste disposal center locations using a hybrid multi-criteria decision-making method. *Sci. Rep.* **13**, 15130 (2023). 37704751 10.1038/s41598-023-42455-w PMC10499883

[35] Kulkarni, N. D., Saha, A. & Kumari, P. Utilizing multicriteria decision-making approach for material selection in hybrid polymer nanocomposites for energy-harvesting applications. *Polym. Compos.* **45**, 6264–6277 (2024).

[36] Kumar, M., Kulkarni, N. D., Saha, A. & Kumari, P. Using multi-criteria decision-making approach for material alternatives in TiO_2_/P(VDF-TrFE)/PDMS based hybrid nanogenerator as a wearable device. *Sens. Actuators A Phys.* **372**, 115331 (2024).

[37] Saha, A., Kulkarni, N. D. & Kumari, P. Development of Bambusa tulda-reinforced different biopolymer matrix green composites and MCDM-based sustainable material selection for automobile applications. *Environ. Dev. Sustain.*10.1007/s10668-023-04327-1 (2023).

[38] Yazdani, M., Zarate, P., Kazimieras Zavadskas, E. & Turskis, Z. A combined compromise solution (CoCoSo) method for multi-criteria decision-making problems. *Manag. Decis.* **57**, 2501–2519 (2019).

[39] Dwivedi, P. P. & Sharma, D. K. Application of Shannon entropy and CoCoSo methods in selection of the most appropriate engineering sustainability components. *Clean. Mater.* **5**, 100118 (2022).

[40] Ghasemi, K., Behzadfar, M., Borhani, K. & Nouri, Z. Geographic information system based combined compromise solution (CoCoSo) method for exploring the Spatial justice of accessing urban green spaces, a comparative study of district 22 of Tehran. *Ecol. Ind.* **144**, 109455 (2022).

[41] Jafari, M. & Naghdi, S. N. Integrated knowledge management in the supply chain: Assessment of knowledge adoption solutions through a comprehensive CoCoSo method under uncertainty. *J. Ind. Inf. Integr.* **39**, 100581 (2024).

[42] Erdal, H., Kurtay, K. G., Dagistanli, H. A. & Altundas, A. Evaluation of anti-Tank guided missiles: An integrated fuzzy entropy and fuzzy CoCoSo multi criteria methodology using technical and simulation data. *Appl. Soft Comput.* **137**, 110145 (2023).

[43] Peng, X., Zhang, X. & Luo, Z. Pythagorean fuzzy MCDM method based on CoCoSo and CRITIC with score function for 5G industry evaluation. *Artif. Intell. Rev.* **53**, 3813–3847 (2020).

[44] Negara, R. M., Syambas, N. R. & Mulyana, E. CRITIC-CoCoSo-based caching placement strategy using multi-criteria decision method for efficient content distribution in named data networking. *J. King Saud Univ. Comput. Inform. Sci.* **35**, 101714 (2023).

[45] Gou, C. An integrated CoCoSo-CRITIC-based decision-making framework for quality evaluation of innovation and entrepreneurship education in vocational colleges with intuitionistic fuzzy information. *Math. Probl. Eng.* **2022**, 6071276 (2022).

[46] Nguyen, V. T. & Chaysiri, R. CRITIC-CoCoSo model application in hybrid solar-wind energy plant location selection problem: A case study in Vietnam. *Energ. Eng.* **122**, 515–536 (2025).

[47] Soni, A., Chakraborty, S., Das, P. K. & Saha, A. K. Materials selection of reinforced sustainable composites by recycling waste plastics and agro-waste: An integrated multi-criteria decision making approach. *Constr. Build. Mater.* **348**, 128608 (2022).

[48] Srivastava, S., Tripathi, A. & Arora, N. Multi-criteria decision making (MCDM) in diverse domains of education: a comprehensive bibliometric analysis for research directions. *Int. J. Syst. Assur. Eng. Manag.*10.1007/s13198-024-02332-9 (2024).

[49] Rishabh, R. & Das, K. N. A critical review on metaheuristic algorithms based multi-criteria decision-making approaches and applications. *Arch. Comput. Methods Eng.*10.1007/s11831-024-10165-9 (2024).

[50] Jakab, S. K., Singh, T., Fekete, I. & Lendvai, L. Agricultural by-product filled poly(lactic acid) biocomposites with enhanced biodegradability: The effect of flax seed meal and rapeseed straw. *Compos. Part. C Open Access* **14**, 100464 (2024).

[51] Diakoulaki, D., Mavrotas, G. & Papayannakis, L. Determining objective weights in multiple criteria problems: The critic method. *Comput. Oper. Res.* **22**, 763–770 (1995).

[52] Singh, T. An integrated multicriteria decision making framework for the selection of waste cement dust filled automotive brake friction composites. *Sci. Rep.* **14**, 6817 (2024). 38514706 10.1038/s41598-023-46385-5 PMC11176197

[53] Alhamad, H. E. & Al-Mandil, S. M. From intuition to optimization: A hybrid FAHP-MAUT model for informed R&D investment decision in mining. *Min. Metall. Explor.* **41**, 2457–2478 (2024).

[54] Khan, S. A., Siddiqui, M. A., Khan, Z. A., Asjad, M. & Husain, S. Numerical investigation and implementation of the Taguchi based entropy-ROV method for optimization of the operating and geometrical parameters during natural convection of hybrid nanofluid in annuli. *Int. J. Therm. Sci.* **172**, 107317 (2022).

[55] Singh, T. Entropy weighted WASPAS and MACBETH approaches for optimizing the performance of solar water heating system. *Case Stud. Therm. Eng.* **53**, 103922 (2024).

[56] Borysiuk, P. et al. Influence of a bark-filler on the properties of PLA biocomposites. *J. Mater. Sci.* **56**, 9196–9208 (2021).

[57] Mengeloglu, F. & Karakus, K. Thermal degradation, mechanical properties and morphology of wheat straw flour filled recycled thermoplastic composites. *Sensors* **8**, 500–519 (2008). 27879719 10.3390/s8010500 PMC3681138

[58] Väisänen, T., Haapala, A., Lappalainen, R. & Tomppo, L. Utilization of agricultural and forest industry waste and residues in natural fiber-polymer composites: A review. *Waste Manag.* **54**, 62–73 (2016). 27184447 10.1016/j.wasman.2016.04.037

[59] Robledo-Ortíz, J. R., Campo, A. S. M., Blackaller, J. A., González-López, M. E. & Fonseca, A. A. P. Valorization of sugarcane straw for the development of sustainable biopolymer-based composites. *Polymers* **13**, 3335 (2021). 34641150 10.3390/polym13193335 PMC8512035

[60] Das, O., Sarmah, A. K. & Bhattacharyya, D. Biocomposites from waste derived Biochars: Mechanical, thermal, chemical, and morphological properties. *Waste Manag.* **49**, 560–570 (2016). 26724232 10.1016/j.wasman.2015.12.007

[61] Kuciel, S., Mazur, K. & Hebda, M. The influence of wood and basalt fibres on mechanical, thermal and hydrothermal properties of PLA composites. *J. Polym. Environ.* **28**, 1204–1215 (2020).

[62] Pokhriyal, M. & Rakesh, P. K. Processing and characterization of novel Himalayacalamus falconeri fiber reinforced biodegradable composites. *Biomass Convers. Biorefinery*. **14**, 21245–21260 (2024).

[63] Chaudhary, V. et al. Development and mechanical characterization of PLA composites reinforced with jute and nettle bio fibers. *Biomass Convers. Biorefinery*. 10.1007/s13399-023-05183-9 (2023).

[64] Azka, M. A., Sapuan, S. M., Abral, H., Zainudin, E. S. & Aziz, F. A. An examination of recent research of water absorption behavior of natural fiber reinforced polylactic acid (PLA) composites: A review. *Int. J. Biol. Macromol.* **268**, 131845 (2024). 38677695 10.1016/j.ijbiomac.2024.131845

[65] Manral, A., Bajpai, P. K. & Khanna, P. Effect of non-acidic chemical treatment of Kenaf fiber on biodegradation and thermal degradation of Kenaf/PLA green composite laminates. *Biomass Convers. Biorefinery*. 10.1007/s13399-023-05010-1 (2023).

